# Non-coding RNAs: Important participants in cardiac fibrosis

**DOI:** 10.3389/fcvm.2022.937995

**Published:** 2022-07-28

**Authors:** Yiheng Dong, Naling Peng, Lini Dong, Shengyu Tan, Xiangyu Zhang

**Affiliations:** Department of Geriatrics, The Second Xiangya Hospital, Central South University, Changsha, China

**Keywords:** non-coding RNA, microRNA, long non-coding RNA, cardiac fibrosis, biomarker, cardiac remodeling

## Abstract

Cardiac remodeling is a pathophysiological process activated by diverse cardiac stress, which impairs cardiac function and leads to adverse clinical outcome. This remodeling partly attributes to cardiac fibrosis, which is a result of differentiation of cardiac fibroblasts to myofibroblasts and the production of excessive extracellular matrix within the myocardium. Non-coding RNAs mainly include microRNAs and long non-coding RNAs. These non-coding RNAs have been proved to have a profound impact on biological behaviors of various cardiac cell types and play a pivotal role in the development of cardiac fibrosis. This review aims to summarize the role of microRNAs and long non-coding RNAs in cardiac fibrosis associated with pressure overload, ischemia, diabetes mellitus, aging, atrial fibrillation and heart transplantation, meanwhile shed light on the diagnostic and therapeutic potential of non-coding RNAs for cardiac fibrosis.

## Introduction

The cardiac extracellular matrix (ECM) predominantly consists of collagen I and collagen III. Collagen I provides tensile strength and collagen III maintains the elasticity. ECM also contains various cytokines and proteases, which have diverse effects on biological characteristics of cardiac cells and regulate ECM structure. There are also a large number of resident cardiac fibroblasts (CFs, the main source of ECM), pericytes, smooth muscle cells, and small populations of immune cells in cardiac interstitium, which play an important role in maintaining homeostasis of ECM. ECM constitutes scaffold of the heart and provides microenvironmental support for cardiomyocytes (CMs).

Normal construction of ECM is vital to maintain ventricular systolic and diastolic function. Under physiological conditions, the ECM achieves a balanced turnover through degradation and synthesis. Nevertheless, this balance often gets disturbed under various pathological conditions, such as injury, pressure overloading, and metabolic disorder. Limited cardiac fibrosis, namely, adaptive remodeling, is beneficial for normal structure of the heart. Aberrant cardiac fibrosis, namely, maladaptive remodeling, increases the stiffness of ventricle and impairs the transmission of mechanical force, which eventually develops into heart failure (HF) with either preserved or reduced ejection fraction (1).

Based on current studies, multiple signaling pathways have been identified to participate in the pathological process of cardiac fibrosis. TGF-β superfamily, renin-angiotensin-aldosterone system, and adrenergic signaling have been extensively investigated. Endothelin, inflammatory cascades, oxidative stress, mitogen-activated protein kinase (MAPKs), and Wnt signaling also participate in this process (2).

Three-quarters of the human genome can be transcribed into RNA, while protein-coding genes represent only 2% of the whole genome (3) and residual transcripts are non-coding RNAs (ncRNAs). These ncRNAs transcripts mainly include microRNAs (miRNAs) and long ncRNAs (lncRNAs). MiRNAs are a class of small (≈22 nucleotides long) ncRNAs that induce gene silence by binding to the 3′ untranslated regions of target mRNAs in most cases. Interaction of miRNAs with 5′ untranslated regions or coding region of mRNAs also exists in a few cases (4). LncRNAs are defined as RNAs longer than 200 nucleotides, which are not translated into functional proteins. Regulatory mechanisms of LncRNAs on genes expression are complex and mediated by their interaction with DNA, RNA, and protein (5). Many studies have proved that ncRNAs play a crucial role in various cardiac diseases, including cardiac fibrosis. This review will summarize the role of miRNAs and lncRNAs in cardiac fibrosis, meanwhile discuss the potential role of these ncRNAs as novel biomarkers and therapeutic candidates.

## Non-coding RNAs in cardiac fibrosis

### Pressure overload-related cardiac fibrosis

Pressure overload-related cardiac fibrosis characterized by interstitial fibrosis or perivascular fibrosis is primarily induced by hypertension or valvular stenosis because of increased afterload in human. Transverse aortic constriction (TAC) in mouse is a recognized method to simulate left ventricular (LV) pressure overload in human. Angiotensin II has been also widely used to produce hypertension in mice.

#### MicroRNAs

##### MiR-21

MiR-21 is a cardiac fibrosis-related ncRNA which have got in-depth investigations in recent years. Increased levels of miR-21 in plasma and cardiac tissue were found in patients suffering from hypertension, aortic valve stenosis, and HF compared with healthy controls (6–8). By *in situ* hybridization, miR-21 expression was detected predominantly in CFs rather than CMs (6). In established pressure overload murine model subjected to TAC, miR-21 could protect CFs from apoptosis and promote them survival (6). By comparing wild type with osteopontin-knockout mice subjected to chronic angiotensin II infusion, osteopontin was identified as an upstream molecule of miR-21; Phosphatase and Tensin Homologue/*Drosophila* Mothers against Decapentaplegic Protein 7 (PTEN/SMAD7) were downstream targets (7). MiR-21 also regulates the transformation of other cell types to CFs in myocardial tissues. In one study, miR-21 was found to promote bone marrow fibroblast progenitor cells homing and trans-differentiation into myofibroblasts in pressure-overloaded myocardium, which could be inhibited by IL-10 (9). MiR-21 was also expressed in macrophages in TAC murine models, even far higher than in CFs; specific genetic deletion of miR-21 in macrophages induced their polarization toward an anti-inflammatory M2-like phenotype rather than pro-inflammatory M1-like phenotype, which mitigated cardiac fibrosis and improved heart function (10). Through ligand-receptor–pairing analysis and an *in vitro* experiment, M1-like phenotype macrophages could activate differentiation of CFs to myofibroblasts in a paracrine manner (10).

##### MiR-30

MiR-30 family is composed of miR-30a, miR-30b, miR-30c, miR-30d, and miR-30e. Due to differences in seed sequences, they perform diverse biological functions *via* targeting different molecules. MiR-30 family was proved to have cardiac protective function. In hypertension- or TAC-induced LV hypertrophy animal model, significant downregulation of miR-30c in CFs caused high expression of connective tissue growth factor (CTGF) and excessive cardiac fibrosis (11). In another experiment performed on mice subjected to TAC, miR-30d was demonstrated to be essential for preventing maladaptive cardiac remodeling (12).

##### MiR-25

Ion channel plays an important role in cellular physiological function. MiR-25 could regulate cardiac fibrosis through altering expression of ion channel. The calcium-transporting ATPase SERCA2a primarily mediates Ca^2+^ uptake during excitation-contraction coupling in CMs. MiR-25 was a suppressor of SERCA2a and pathologically upregulated in myocardial tissues from patients with severe HF (13). *In situ* hybridization revealed that miR-25 was expressed primarily in CMs rather than CFs or vascular endothelial cells in mouse subjected to TAC (13). Anti-miR-25 improved cardiac contractility and showed anti-fibrotic effects, which were confirmed by an elevated level of SMAD7 and a decreased tendency of α-smooth muscle actin (α-SMA), a marker of fibrosis (14).

##### MiR-26

In spontaneously hypertensive rats, the plasma and myocardial miR-26a levels were inversely associated with blood pressure and cardiac fibrosis (15). Upregulation of miR-26a can ameliorate hypertension-induced cardiac fibrosis through directly inhibiting the expression of fibrosis-related genes, such as Enhancer of Zeste Homolog 2 (EZH2), CTGF, and SMAD4 (15).

##### MiR-133

MiR-133 is exclusively expressed in CMs and has a positive influence on cardiac fibrosis *via* multiple signaling pathways. In a follow-up study, patients with inflammatory cardiomyopathy and preserved LV function at study entry showed higher expression of miR-133 than patients with reduced LV function (16). A high level of miR-133 was associated with less fibrosis and myocyte necrosis (16). In pressure overload-induced LV hypertrophy, miR-133 was significantly downregulated, which resulted in upregulation of CTGF and excessive ECM deposition (11). Subsequent experiment reported that downregulation of miR-133 was correlated to histone deacetylase (HDAC) (17). In addition, overexpression of miR-133 could prevent CMs from apoptosis in mouse subjected to TAC through directly targeting β-adrenergic receptors (βARs) as well as several other components of the βARs signaling cascade, which contributed to less fibrosis (18).

##### MiR-29

MiR-29 family comprises three members, namely, miR-29a, miR-29b, and miR-29c, which are derived from two bicistronic miRNA clusters. MiR-29a is co-expressed with miR-29b1, whereas miR-29c is co-expressed with miR-29b2. All family members share a conserved seed region (19). In *in vitro* experiments, miR-29 has been demonstrated to exert anti-fibrotic function and inhibit the proliferation of CFs and the expression of fibrosis-related genes *via* targeting cell cycle related protein cyclin-dependent kinases (CDK2) and vascular endothelial growth factor-A (VEGF-A)/MAPK signaling (20, 21). However, as per *in vivo* studies, miR-29 family seems to exert complex functions. In a hypertensive myocardial fibrosis model induced by angiotensin II, miR-29b was significantly downregulated in heart tissues; recovery of miR-29b expression was sufficient to prevent or rescue hypertensive myocardial fibrosis induced by angiotensin II, and partly attenuated reduction of cardiac function *via* repressing TGF-β/SMAD3 as well as MAPK pathways (22). After angiotensin II infusion, miR-29a/b1 knockdown mouse exhibited more obvious diastolic dysfunction, severer cardiac fibrosis and systemic hypertension when compared with wild type (23). These studies demonstrated the anti-fibrotic effect of miR-29. But contradictory results were obtained in another study, in which systemic miR-29 knockout (miR-29a/b1 or miR-29b2/c) protected mouse from cardiac hypertrophy and interstitial fibrosis caused by TAC (24). The controversial results in different studies might be caused by the following reasons: (1) Different studies utilized distinct animal models. Compared with angiotensin II, TAC induces the onset of pressure overload more quickly, which might involve different pathophysiological processes. (2) The precise cell source of miR-29 remains unclear. It had been validated that CFs was the main source of miR-29 in some experiments (19), whereas in other studies, miR-29 was mainly derived from CMs (24). MiR-29 exerts antifibrotic functions in CFs but pro-hypertrophic functions in CMs. MiR-29 significantly increased within 48 h and downregulated to baseline level 21 days after TAC in CMs; temporal expression pattern in CFs was unknown (24). Therefore, it needs further studies to explore the exact effects of miR-29 on cardiac fibrosis. In addition, cell-type-specific expression characteristics of miR-29 over time and potential impacts of disease models on miR-29 expression should be investigated.

##### MiR-320

MiR-320 has similar cell-type-specific functions like miR-29. In TAC mouse models, miR-320 was upregulated in CMs but downregulated in CFs. The increased expression of miR-320 in CMs led to deterioration of cardiac function through inhibiting Pleckstrin Homology Domain Containing M3 (PLEKHM3), whereas the decreased expression of miR-320 in CFs caused myocardial fibrosis *via* derepressing interferon-induced transmembrane protein 1 (IFITM1) (25). Dysregulation of miR-320 between different cell types was mediated by a cluster of cell-type-specific transcriptional factors and distinct expression patterns of argonaute 2, which was required for the stability of mature miR-320 (25). Recovery of miR-320 expression to normal levels in CMs and CFs, respectively, seems to be prospective therapeutics for HF.

##### MiR-214

MiR-214 is a highly conserved miRNA among vertebrates and is expressed in multiple tissues. In mouse, miR-214 is encoded by an opposite strand of Dynamin3 gene on chromosome 1 (26). In the heart of pressure overload mouse models induced by TAC, miR-214-3p was remarkably downregulated; restoring its expression could inhibit the proliferation and differentiation of CFs and attenuate cardiac fibrosis (27). But another study found profibrotic effects of miR-214 (28). In this study, miR-214 derived from T cells was significantly increased and positively correlated with perivascular fibrosis in hypertensive mouse models induced by angiotensin II, and plasm miR-214 in patients with hypertension was higher than in controls and directly related to pulse wave velocity, a marker for vascular sclerosis (28). MiR-214 was downregulated in TAC mouse models but upregulated in patients with hypertension. The discrepancy between animal models and patients with hypertension needs further investigation.

##### MiR-212/132

MiR-212/132 family originates mainly from CMs. The upregulation of miR-212/132 has been found in defective heart of mouse and human (29). An *in vitro* experiment has demonstrated that the expression of miR-212/132 in CMs could be induced under hypertrophic stimulus such as angiotensin II, insulin-like growth factor-1, and phenylephrine/isoprenaline (29). *In vivo*, CMs-specific overexpression of miR-212/132 reduced life expectancy of mouse, which exhibited severe HF symptoms as well as dramatically increased atrial natriuretic peptide (ANP) and brain natriuretic peptide (BNP). Compared with wild type, attenuation of cardiac dysfunction and decrease of cardiac fibrosis were observed in mouse subjected to TAC with CMs-knockout of miR-212/132 (29). Foxo3 is the direct downstream target of miR-212/132. Inhibition of Foxo3 by miR-212/132 leads to downregulation of autophagy level and upregulation of calcineurin/NFAT signaling, as well as deterioration of cardiac function (29). Moreover, expression of miR-132-3p was sufficient to promote phenotypic transition of human fibroblasts and deposition of ECM *via* inhibiting Foxo3 (30). But, in another study, the same authors obtained different results and found that upregulation of miR-212/132 may have protective effects during pressure overload (31). The expression of Methyl-CpG–binding Protein 2 (MeCP2), which was associated with mitochondrial function, was inhibited after TAC; CMs-specific loss of MeCP2 in mouse subjected to TAC attenuated maladaptive myocardial remodeling and facilitated recovery of cardiac dysfunction when removal of the aortic constriction compared with wild type (31). Further analysis found inhibition of MeCP2 after TAC was mediated by activation of adrenergic signaling and upregulation of miR-212/132 (31). In summary, upregulation of miR-212/132 under pressure overload might be a compensatory mechanism, which provides benefit to normal cardiac function in the early period of disease through promoting CMs hypertrophy and limited interstitial fibrosis, and maintaining energy metabolism homeostasis. Nevertheless, continuous upregulation of miR-212/132, when pressure overload sustains for a long time, will lead to maladaptive myocardial remodeling.

The roles of miR-Let7i (32), miR-1954 (33), miR-378 (34), miR-221/222 (35), miR-199a (36), miR-99b-3p (37), miR-125b (38), and miR130a (39) in cardiac fibrosis are displayed in Table 1.

**TABLE 1:** Effects of miRNAs on cardiac fibrosis.

MiRNAs	Cell model	Animal model	Changes in model	Targets	Effects on fibrosis	References
**Pressure overload-related cardiac fibrosis**						
mmu-miR-21	CFs, bone marrow fibroblast progenitor cells (BM-FPC) treated with TGF-β, macrophages	TAC or treated with angiotensin II infusion	Upregulated	Sprouty1, PTEN/SMAD7	Promote fibrosis. Increase survival of CFs, BM-FPC trans-differentiation to CFs, expression of α-SMA and CTGF	(6, 7, 9, 10)
mmu-miR-30c and mmu-miR-30d	CFs and CMs	TAC	Downregulated	CTGF	Inhibit fibrosis. Decrease expression of CTGF, collagen I and III, fibronectin, and α-SMA	(11, 12)
mmu-miR-25	CMs	TAC	Upregulated	SERCA2a	Promote fibrosis. Increase expression of α-SMA	(13, 14)
mmu-miR-26a	CFs treated with angiotensin II	Spontaneously hypertensive rat	Downregulated	EZH2, CTGF, SMAD4	Inhibit fibrosis. Decrease proliferation of CFs, expression of CTGF, collagen I and III, MMP2	(15)
mmu-miR-133	CMs and CFs	TAC	Downregulated	CTGF, βARs	Inhibit fibrosis. Decrease expression of CTGF and prevent CMs from apoptosis	(11, 17, 18)
mmu-miR-29	CFs	Treated with angiotensin II infusion	Downregulated	CDK2, VEGF-A/MAPK signaling, TGF-β, PGC1α	Inhibit fibrosis. Decrease fibrotic area	(20–23)
	CMs	TAC	Upregulated in CMs	Wnt signaling	Promote fibrosis. Increase hypertrophy of CMs and fibrotic area	(24)
mmu-miR-320	CFs and CMs	TAC	Downregulated in CFs. Upregulated in CMs	IFITM1 in CFs, PLEKHM3 in CMs	Inhibit fibrosis in CFs. Decrease expression of collagen I and fibronectin.	(25)
mmu-miR-214-3p	CFs	TAC	Downregulated	NOD-like Receptor Family CARD Domain Containing 5	Inhibit fibrosis. Decrease expression of collagen I and α-SMA	(27)
mmu-miR-212/132	CMs, CFs	TAC	Upregulated	FoxO3 and MeCP2	Promote fibrosis. Increase hypertrophy of CMs and activation of CFs, expression of collagen I and CTGF	(29–31)
mmu-miR-Let7i	CFs treated with angiotensin II	Treated with angiotensin II	Downregulated	IL-6 and collagen	Inhibit fibrosis. Decrease expression of collagen	(32)
mmu-miR-1954	CFs treated with angiotensin II	Treated with angiotensin II	Downregulated	Thrombospondin 1	Inhibit fibrosis. Decrease expression of collagen	(33)
mmu-miR-378	CMs and CFs	TAC	Downregulated	Mitogen-activated protein kinase kinase 6	Inhibit fibrosis. Decrease fibrotic area, expression of collagen I and III	(34)
mmu-miR-221/222	CFs treated with TGF-β	Treated with angiotensin II	Downregulated	C-Jun N-terminal kinase 1, TGF-β receptor 1 and 2, and ETS proto-oncogene 1	Inhibit fibrosis. Decrease proliferation and activation of CFs and fibrotic area.	(35)
mmu-miR-199a	CFs and CMs	TAC or treated with angiotensin II or isoproterenol	Upregulated	Sirt 1 for miR-199a-5p and SMAD1 for miR-199a-3p	Promote fibrosis. Increase fibrotic area expression of collagen I, III and α-SMA	(36)
mmu-miR-99b-3p	CFs treated with angiotensin II	Treated with angiotensin II	Upregulated	Glycogen synthase kinase-3 beta	Promote fibrosis. Increase fibrotic area, expression of fibronectin, collagen I and α-SMA	(37)
mmu-miR-125b	CFs treated with TGF-β	TAC or treated with angiotensin II	Upregulated	Apelin	Promote fibrosis. Increase proliferation and activation of CFs and fibrotic area	(38)
mmu-miR-130a	CFs treated with angiotensin II	Treated with angiotensin II	Upregulated	Peroxisome proliferator-activated receptor γ	Promote fibrosis. Increase fibrotic area, expression of collagen I, III, CTGF, fibronectin and α-SMA	(39)
**Ischemia-related cardiac fibrosis**						
mmu-miR-30d	CFs and CMs	MI	Upregulated in acute ischemic stress. Downregulated in chronic ischemic stress	mitogen associated protein kinase 4 in CMs, integrin α5 in CFs	Inhibit fibrosis. Decrease apoptosis of CMs, proliferation and activation of CFs	(44)
mmu-miR-26a	CFs and CMs	MI	Downregulated	ATM/p53	Inhibit fibrosis. Decrease apoptosis of CMs, expression of collagen I and CTGF	(45)
mmu-miR-150	Monocytes, CFs and CMs	MI	Downregulated	CXCR4 in monocytes; SPRR1a, egr2 and p2 × 7r in CMs; Hoxa4 in CFs	Inhibit fibrosis. Decrease accumulation of monocytes to myocardium, apoptosis of CMs, and fibrotic area	(47–50)
mmu-miR-144	Didn’t use	MI	Downregulated	mTOR	Inhibit fibrosis. Decrease fibrotic area, expression of MMP	(51, 52)
mmu-miR-29	CFs	MI	Downregulated		Inhibit fibrosis. Decrease expression of collagen	(19)
mmu-miR-214	CMs	MI	Upregulated	Sodium/calcium exchanger 1 and CTRP9 in CMs	Unclear.	(26, 53)
hsa-miR-132	Human pericyte progenitor cells and CFs	MI	Upregulated in pericyte progenitor treated with hypoxia/starvation	MeCP2	Inhibit fibrosis. Decrease proliferation and differentiation of CFs.	(54)
mmu-miR-433	CFs treated with TGF-β	MI	Upregulated	AZIN1, JNK1	Promote fibrosis. Increase fibrotic area, proliferation and activation of CFs, expression of CTGF, collagen I and III, and α-SMA	(55)
mmu-miR-384-5p	CFs treated with TGF-β	IR	Downregulated	Fzd1 and 2, TGF-β-R1 and Lrp6	Inhibit fibrosis. Decrease fibrotic area, activation of CFs, expression of collagen I and α-SMA	(56)
mmu-miR-370	CFs	MI	Downregulated	TGF-β-R1	Inhibit fibrosis. Decrease of CTGF and α-SMA	(57)
mmu-miR-146-5p	CFs, macrophages and endothelial cells	MI	Upregulated	Interleukin 1 receptor associated kinase 1 and Carcinoembryonic antigen related cell adhesion molecule 1	Promote fibrosis. Increase proliferation and activation of CFs, and fibrotic area	(58)
mmu-miR-143-3p	CFs treated with TGF-β	MI	Upregulated	Sprouty 3	Promote fibrosis. Increase proliferation and activation of CFs, and fibrotic area	(59)
**Diabetes mellitus-related cardiac fibrosis**						
mmu-miR-21	CFs and endothelial cells treated with high glucose	Streptozotocin-induced diabetic model	Upregulated	DUSP 8	Promote fibrosis.	(70, 71)
**Age-related cardiac fibrosis**						
hsa-1468-3p	CFs with TGF-β		Upregulated	DUSP 1, 6, 8 and p53/p16	Promote fibrosis. Increase senescence-associated b-galactosidase activity, expression of collagen I, CTGF and periostin	(75)
**Atrial fibrosis**						
mmu-miR-21	CFs	Mouse model	Upregulated	Sprouty1	Promote fibrosis. Increase fibrotic area and spontaneous atrial fibrillation in older age.	(77)
cfa-miR-29	Fibroblasts	Canine atrial fibrillation models induced by ventricular tachypacing, mouse model	Downregulated	ECM-genes, including collagen 1A1, collagen 3A1, and fibrillin	Inhibit fibrosis. Decrease expression of collagen I and III, and fibrillin	(78)
cfa-miR-26	Atrial fibroblasts, CFs	Dogs with ventricular tachypacing-induced congestive HF	Downregulated	KCNJ2/TRPC3	Inhibit fibrosis. Decrease proliferation of CFs	(79–81)
**Heart transplantation-related cardiac fibrosis**						
mmu-miR-21	RAW 264.7 cells (murine monocytic cell line)	Allogeneic and isogeneic murine heart transplantation	upregulated	PTEN/AP-1	Promote fibrosis. Increase fibrocyte accumulation in myocardium, expression of α-SMA and vimentin	(82)

#### Long non-coding RNAs

Limited evidences display the role of lncRNAs in pressure overload-related fibrosis. In TAC murine models, lncRNA maternally expressed gene 3 (MEG3) was found to be enriched mainly in CFs in comparison with other cardiac cell types and its transcript was upregulated at the initial phase of cardiac remodeling (40). MEG3 could interact with P53 and consequently promote the binding of P53 to the promoter of matrix metalloprotease-2 (MMP2), leading to increased MMP2 expression and MMP2-mediated deposition of ECM; silencing of MEG3 could prevent the development of cardiac fibrosis and hypertrophy (40). Cardiac Hypertrophy–Associated Transcript (CHAST) is a pro-hypertrophic lncRNA induced by NFAT. It can regulate hypertrophic genes through inhibiting Pleckstrin Homology Domain–containing Protein Family M Member 1 (PLEKHM1) and cardiac fibrosis *via* paracrine pathways (41). Myosin Heavy-chain-associated RNA Transcripts (MHRT) is a cardioprotective lncRNA enriched in CMs, which decreases under various pathological stress conditions. MHRT can bind to the helicase domain of Brg1 and prevent Brg1 from recognizing its genomic DNA targets. Restoring MHRT to the normal level protected the heart from hypertrophy and cardiac fibrosis after TAC (42).

### Ischemia-related cardiac fibrosis

Ischemia-related cardiac fibrosis belongs to replacement fibrosis caused by necrotic injury of CMs.

#### MicroRNAs

##### MiR-21

In myocardial infarction (MI) murine model, miR-21 was upregulated and promoted the expression of MMP2 *via* a PTEN pathway in CFs (43), which probably influenced the turnover of ECM.

##### MiR-30

MiR-30 plays an important role in ischemia or MI-related cardiac fibrosis. After acute ischemic stress, miR-30d was significantly upregulated in CMs and CMs-derived extracellular vesicles (EVs). Increase of miR-30d reduced CMs apoptosis and CFs activation through EVs-mediated paracrine signaling (44). However, in chronic ischemic HF, decreased expression of miR-30d induced adverse cardiac remodeling, which was validated both in mouse and in humans (44). The exact mechanisms of downregulation of miR-30d in chronic ischemic heart disease remain unknown and need further investigation.

##### MiR-26

In patients with ST−elevation MI, expression of miR-26a was downregulated (45). Transfection H9c2 cells with miR-26a could reduce apoptosis. Overexpression of this miRNA *in vivo* could decrease the levels of collagen I as well as CTGF and improve cardiac function *via* regulating ataxia–telangiectasia mutated (ATM)/p53 signaling (45).

##### MiR-150

Low circulating levels of miR-150 were associated with LV remodeling after first ST-elevation acute MI (46). During acute MI, expression of miR-150 was remarkably decreased; restoration of its expression could improve cardiac function, reduce infarct areas, and attenuate fibrosis (47–50). Mechanically, miR-150 could reduce monocyte accumulation to myocardium by inhibiting CXCR4, decrease CMs apoptosis through repressing pro-apoptotic genes small proline–rich protein 1a (SPRR1a), egr2 and p2×7r, and prevent activation of CFs *via* downregulating pro-fibrotic Homeobox a4 (Hoxa4) (47–50).

##### MiR-144

MiR-144 was significantly decreased during acute MI. MiR-144-knockout aggravated HF after MI. Intravenous injection of miR-144 mimics could sufficiently attenuate fibrosis and improve ventricular function (51). Similarly, remote ischemic preconditioning induced by cycles of transient limb ischemia and reperfusion (IR) could enhance expression of miR-144 and attenuate cardiac dysfunction induced by IR injury (52).

##### MiR-29

During MI, expression of miR-29 was decreased in the border zone adjacent to the infarct and remote areas, which was significantly related to the upregulation of fibrosis-related genes (19). Inhibition of miR-29b by cholesterol-modified oligonucleotides could lead to moderate increment of collagen expression (19). Thus, miR-29 family seems to have an anti-fibrotic function in MI.

##### MiR-214

In ischemia myocardial disease, miR-214 plays a complex role in regulation of cardiac function. During IR, miR-214 was remarkably upregulated in wild-type mouse and death of myocytes caused by calcium overload was attenuated (26). Compared with wild type, ligation of the left anterior descending coronary artery in miR-214-knockout mouse resulted in a significant increase in mortality accompanied by more obvious reduction in cardiac function and more extensive myocardial fibrosis (26). A study found that chronic intermittent hypoxia exposure during MI period in mouse led to significant upregulation of miR-214-3p and then reduction of cardio-protective factors C1q tumor necrosis factor-related protein-9 (CTRP9). Suppression of miR-214 by antagomiR-214-3p can prevent cardiac hypertrophy and myocardial remodeling (53). Possible reasons for the contradiction of these two studies are listed as follows: (1) Different strands of the same miRNA have different functions. In the former study, systemic knockout of miR-214 was performed, which means both miR-214-3p and 5p were deleted; while in the latter study, only miR-214-3p was knockout, which might have no influence on the expression of miR-214-5p. (2) The former study indicated that miR-214 exerted cardio-protective functions *via* myocytes. But the latter study suggested that miR-214-3p caused maladaptive remodeling through both myocytes and fibroblasts, the potential functions of miR-214-5p were not investigated. (3) MI with or without chronic intermittent hypoxia exposure may involve distinct pathophysiological process, which might contribute to the controversy of miR-214 functions.

##### MiR-132

MiR-132 seems to have cardio-protective effects during MI. MeCP2 remarkably increased after MI and miR-132 secreted from human pericyte progenitor cells could inhibit expression of MeCP2 and prevent proliferation and differentiation of CFs, finally reduce interstitial fibrosis (54).

The roles of miR-433(55), miR-384-5p (56), miR-370 (57), miR-146-5p (58), and miR-143-3p (59) in cardiac fibrosis are displayed in Table 1.

#### Long non-coding RNAs

Comparing expression profiles of lncRNAs in ischemic cardiomyopathy with control hearts through bioinformatic technology, 145 lncRNAs were differentially expressed (60) and most of them were correlated with expression of fibrogenic genes such as collagen 3A1, collagen 8A1, and fibronectin. In this study, five highly conserved and CFs-enriched lncRNAs were selected to identify their function on cardiac fibrosis; knockdown of these lncRNAs, respectively, reduced the expression of fibrogenic genes. Another study found that ischemia promoted EVs secretion from CMs (61). These EVs containing specific lncRNAs were transferred to fibroblasts and led to profibrotic phenotype. Neat1 was one of the EV-enriched lncRNAs and was regulated by P53 and hypoxia-inducible factors-2A (HIF-2A). Silencing of Neat1 could induce apoptosis and inhibit the expression of pro-fibrotic genes.

LncRNA H19 is enriched in the heart and skeletal muscle, which is mainly expressed during embryonic development and repressed after birth. After MI, HIF-1α induced re-expression of H19 in the peri-infarct area (62). Overexpression of H19 resulted in severe cardiac dilation; meanwhile, significant increase of the infarct area and fibrosis was observed. H19 could bind to YB-1, a repressor of collagen I gene, and destroyed the interaction between YB-1 and collagen I promoter. Wisp2 Super-Enhancer–Associated RNA (WISPER) was a cardiac enhancer-associated lncRNA and its expression predominantly enriched in CFs. Silencing of WISPER *in vitro* using antisense oligonucleotides could result in increased apoptosis of CFs and inhibition of ECM-related protein coding genes, including collagen, fibronectin, and TGF-β (63). Therapeutic depletion of WISPER *in vivo* can inhibit cardiac fibrosis and improve heart function after MI. LncRNA NON-MMUT022555, namely Pro-Fibrotic LncRNA (PFL), also markedly increased after MI in mouse and enriched in CFs compared with CMs (64). PFL can directly bind to let-7d, an inhibitory RNA of cardiac fibrosis, in a sequence-specific manner and promote fibroblast-myofibroblast transition. Recently, a novel conserved anti-fibrotic lncRNA was identified, namely, Scaffold Attachment Factor B Interacting LncRNA (SAIL) (65). Expression of SAIL was decreased in cardiac fibrotic tissue and activated CFs. Knockdown of SAIL promoted CFs proliferation and collagen production after MI. SAIL could directly bind to scaffold attachment factor B (SAFB) through 23 conserved nucleotide sequences and prevent interaction between SAFB and RNA polymerase II, thereby decreasing transcription of fibrosis-related genes. Some other lncRNAs were also dysregulated after MI, including cardiac fibroblast-associated transcript (CFAST) (66), metastasis−associated lung adenocarcinoma transcript 1 (MALAT1) (67) lncRNA-30245 (68), and lncRNA AK137033 (69). The functions of these lncRNAs are summarized in Table 2.

**TABLE 2:** Effects of lncRNAs on cardiac fibrosis.

LncRNAs	Cell model	Animal model	Changes in model	Targets	Effects on fibrosis	References
**Pressure overload-related cardiac fibrosis**						
mmu-lncRNA MEG3	CFs treated with TGF-β1	TAC	Upregulated	P53/MMP2	Promote fibrosis. Increase fibrotic area, expression of CTGF	(40)
mmu-lncRNA CHAST	CMs treated with phenylephrine and isoproterenol	TAC	Upregulated	Plekhm1	Promote fibrosis. Increase fibrotic area, expression of CTGF	(41)
mmu-lncRNA MHRT		TAC	Downregulated	Brg1	Inhibit fibrosis, Decrease fibrotic area	(42)
**Myocardial infarction-related cardiac fibrosis**						
mmu-lncRNA H19	CFs under hypoxia	MI	Upregulated	YB-1	Promote fibrosis. Increase fibrotic area, expression of α-SMA, periostin, vimentin and collagen I	(62)
mmu-lncRNA WISPER	CFs	MI	Upregulated	TIA1-related protein	Promote fibrosis. Increase fibrotic area, expression of α-SMA, collagen I, collagen III and fibronectin	(63)
mmu-lncRNA PFL	CFs treated with TGF-β or angiotensin II	MI	Upregulated	Let-7d	Promote fibrosis. Increase fibrotic area, expression of collagen I, CTGF, fibronectin and α-SMA	(64)
mmu-lncRNA SAIL	CFs treated with TGF-β or angiotensin I	MI	Downregulated	SAFB	Inhibit fibrosis. Decrease fibrotic area, expression of collagen I and III	(65)
mmu-lncRNA CFAST	CFs	MI	Upregulated	coactosin-like 1	Promote fibrosis. Increase fibrotic area, expression of collagen, fibronectin and α-SMA	(66)
mmu-lncRNA MALAT1	CFs treated with angiotensin II	MI	Upregulated	miR-145	Promote fibrosis. Increase fibrotic area, expression of collagen I and III, α-SMA	(67)
mmu-lncRNA 30245	CFs treated with TGF-β	MI	Upregulated	PPAR-γ	Promote fibrosis. Increase fibrotic area, expression of collagen I and III	(68)
mmu-lncRNA AK137033	CFs treated with TGF-β	MI	Upregulated	Secreted frizzled-related protein 2	Promote fibrosis. Increase fibrotic area, expression of collagen I and α-SMA	(69)
**Diabetes mellitus-related cardiac fibrosis**						
mmu-lncRNA Kcnq1ot1	CFs treated with high glucose	Streptozotocin-induced diabetic murine model	Upregulated	miR-214-3p	Promote fibrosis. Increase fibrotic area, expression of collagen I and III	(72)
mmu-lncRNA MIAT	CFs treated with high glucose	Streptozotocin-induced diabetic murine model	Upregulated	miR-214-3p	Promote fibrosis. Increase fibrotic area, expression of collagen I and III	(73)
mmu-lncRNA CRNDE	CFs treated with TGF-β or angiotensin II	Streptozotocin-induced diabetic murine model	Upregulated	Smad3	Inhibit fibrosis. Decrease fibrotic area, expression of collagen I and III, α-SMA	(74)

### Diabetes mellitus-related cardiac fibrosis

The prevalence of metabolic diseases worldwide is increasing, especially diabetes mellitus, which results in an enormous healthy and economic burden. Microangiopathy and metabolic disorders caused by diabetes mellitus could lead to extensive focal necrosis of myocardium, namely, diabetic cardiomyopathy (DCM), which eventually induce cardiac fibrosis, HF, arrhythmia, cardiogenic shock, and sudden death.

#### MicroRNAs

MiR-21 could promote CFs proliferation and differentiation after high glucose treatment *via* dual-specificity phosphatases (DUSP) 8/p38/JNK/SAPK axis (70). In a murine model with type I diabetes mellitus, elevated level of p-p65 increased expression of miR-21, which led to downregulation of SMAD7 and activation of p-SMAD2 and p-SMAD3, and then promoted endothelial−mesenchymal transition from endothelial cells to CFs (71).

#### Long non-coding RNAs

Recently, it has been found that multiple lncRNAs play an important role in DCM development, such as lncRNA KCNQ1 Opposite Strand/Antisense Transcript 1 (Kcnq1ot1), lncRNA Myocardial Infarction-associated Transcript (MIAT), and lncRNA Colorectal Neoplasia Differentially Expressed (CRNDE) (72–74). LncRNA Kcnq1ot1 was significantly upregulated in diabetic myocardial tissues. Acting as a competitive endogenous RNA for miR-214-3p, lncRNA Kcnq1ot1 could regulate inflammation, pyroptosis, and biologic properties of fibroblasts *via* modulating expression of caspase-1 (72). Silencing of Kcnq1ot1 could inhibit fibrosis and improve cardiac function. Overexpression of LncRNA MIAT has been detected in serum of patients with diabetes. MIAT could bind to miR-214-3p and then prevent miR-214-3p-mediated inhibitory effect on IL-17 expression. Knockdown of MIAT alleviated cardiac inflammation and fibrosis, consequently improved ejection fraction (73). CRNDE has high interspecies conservation and is mainly enriched in CFs. High expression of CRNDE was observed in mouse with DCM and could be induced by TGF-β and angiotensin II (74). Further investigation found that CRNDE attenuate cardiac fibrosis *via* SMAD3-Crnde negative feedback. SMAD3 transcription could activate CRNDE, whereas CRNDE can bind to SMAD3 and competitively inhibit interaction between SMAD3 and α-SMA promoter to alleviate cardiac fibrosis (74).

### Age-related cardiac fibrosis

Age is one of the risk factors for HF, which is partly associated with progressive cardiac fibrosis. Proliferation of CFs is the characteristic manifestation of cardiac aging. Collagen accumulation was initiated before the occurrence of atrial and ventricular fibrosis in old people. Compared with young individuals, expression of collagen I was increased but collagen III decreased in the elderly. Evidence demonstrated that miRNAs play a vital role in the development of cardiac fibrosis. A substantial increase in cardiac miR-1468-3p levels was detected in healthy hearts in the elderly and aged sudden cardiac death victims with primary myocardial fibrosis (75). By downregulating DUSP1, 6, and 8, miR-1468-3p can enhance TGF-β/p38 pathway and then promote cardiac fibrosis. Meanwhile, miR-1468-3p was attributed to cardiac aging *via* increment of p53 and p16 expression (75). It has been found that all miR-29 variants greatly increased with age (24, 76). MiR-29 also contributed to cellular senescence and cardiac aging *via* epigenetic mechanisms in mouse (76). In view of the close relationship between miR-29 and fibrosis, it is interesting to explore the function of miR-29 in age-related cardiac fibrosis in future studies.

### Atrial fibrosis

There is a close correlation between atrial fibrosis and atrial fibrillation. A number of evidences support vital roles of miRNA in the development of atrial fibrosis.

#### MiR-21

Increased expression of miR-21 and downregulation of its target Spry1 were observed in left atrial myocardium from patients with atrial fibrillation (77). Angiotensin II could induce upregulation of miR-21 in CFs, which is mediated by Rho-GTPase Rac1, CTGF, and lysyl oxidase (a key enzyme of collagen cross-linking). Inhibition of miR-21 with antagomir-21 *in vivo* can prevent atrial fibrosis (77).

#### MiR-29

In canine atrial fibrillation models induced by ventricular tachypacing, miR-29 family was downregulated in atrial tissues. Nevertheless, expression of fibrosis-related genes was increased significantly, such as in collagen I and collagen III (78). Similar characteristics in plasma or atrial tissues from patients with atrial fibrillation and/or congestive HF were observed (78). Using a miR-29b sponge carried by adeno-associated virus (AAV) 9 in mouse could significantly increase myocardial fibrosis related proteins in mouse (78).

#### MiR-26

Downregulation of miR-26a was observed in cardiac tissues obtained from canines and patients with atrial fibrillation, which attributed to its inhibition by NFAT (79). Decreased miR-26a led to upregulation of KCNJ2 (inward-rectifier K^+^ channels Kir2.1 α-subunit was encoded by KCNJ2) expression and enhanced inward-rectifier K^+^-current both in CMs and CFs, thereby making resting membrane potential hyperpolarized and Ca^2+^-entry increased, which could stimulate CFs proliferation (79, 80). Proliferation of CFs was also enhanced by upregulation of Ca^2+^-permeable Transient Receptor Potential Canonical-3 (TRPC3) channels mediated by inhibition of miR-26 (81).

### Heart transplantation related cardiac fibrosis

Cardiac transplantation is the only choice for patients with end-stage HF. After transplantation, the cardiac function of a considerable number of patients is still declining gradually, partly due to fibrosis caused by ischemia, inflammation, and immunological rejection. NcRNAs also participate in transplantation-related fibrosis. It has been observed that miR-21 significantly increases in patient hearts with immune rejection after transplantation (82). Further study demonstrated fibrocytes, with an important role in fibrosis, mainly derived from monocytes, which might be the source of miR-21 in the context of heart transplantation (82). *In vitro*, overexpression of miR-21 could induce phenotypic conversion of monocytes to fibrocytes and increase the levels of fibrotic markers such as α-SMA *via* inhibiting PTEN and activating activator protein 1 (AP-1). *In vivo* inhibition of miR-21 using antisense oligonucleotides could prevent fibrocytes accumulation in cardiac allografts and fibrosis (82).

**FIGURE 1:**
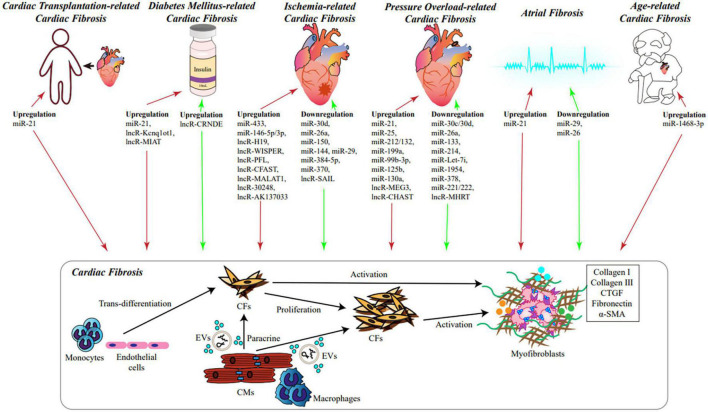
The role of NcRNAs in cardiac fibrosis. During various cardiac diseases and heart aging, significant alteration of cellular transcriptome takes place in cardiac tissues, including ncRNAs, especially miRNAs and lncRNAs, which could influence the process of cardiac fibrosis. Under stimulation, monocytes and endothelial cells can trans-differentiate into CFs; ncRNAs and cellular factors derived from CMs and macrophages could be secreted into EVs or released to extracellular microenvironment, and absorbed by CFs; expression of ncRNAs in CFs can be changed as well. Consequently, CFs would proliferate and differentiate into myofibroblasts *via* different signaling pathways. Myofibroblasts secrete a large amount of ECM, which leads to cardiac fibrosis eventually. Red arrows represent promoting fibrosis, green arrows represent inhibiting fibrosis. ncRNAs, non-coding RNAs; miRNAs, microRNA; lncRNAs, long non-coding RNAs; CMs, cardiomyocytes; CFs, cardiac fibroblasts; EVs, extracellular vesicles; ECM, extracellular matrix.

The complex relationship between ncRNAs and cardiac fibrosis is displayed in Figure 1.

## Non-coding RNAs as diagnostic or prognostic markers for cardiac fibrosis

Multiple cardiac diseases eventually develop into HF accompanied with progressive maladaptive remodeling. The degree of cardiac remodeling is closely related to clinical prognosis. Currently, most of the parameters reflecting remodeling come from imaging technology and are difficult to predict clinical outcome. In view of their correlation with cardiac remodeling, ncRNAs seem to be effective biomarker candidates.

RNA sequencing revealed that there was a significant alteration of ncRNAs in myocardial tissues (83) and blood samples (84) from patients with HF, including fibrosis-related miRNAs. Meanwhile, the expression signatures of these altered ncRNAs can discriminate failing heart from different etiologies (83). Utilizing ROC curve analysis, high specificity and sensitivity of three-miRNAs combination (miR-29b-3p, miR-29c-3p, and miR-451a) correlated with pulmonary capillary wedge pressure (PCWP) get validated (84); early detection and dynamic observation of these miRNAs may predict HF occurrence in the initial phase of cardiac disease. In the context of MI (85) and hypertrophic cardiomyopathy (86), a specific combination of circulating miRNAs has a high predictive value for the presence of cardiac fibrosis. Expression of endomyocardial miR-133a negatively correlated with cardiac fibrosis; subsequent follow-up study revealed that patients with a high expression of miR-133a have low occurrence rate of cardiovascular death, malignant arrhythmia, and hospitalization for HF (16). Compared with traditional risk factors, lower circulating miR-132 could improve risk prediction for HF re-hospitalization (87). MiR-150-5p was significantly downregulated in patients with advanced HF and correlated with maladaptive remodeling, disease severity, and prognosis (88). MiR-150 was also superior to N-terminal pro-BNP to predict remodeling (46). It has been demonstrated that miR-29a was upregulated in the serum of patients with hypertrophic cardiomyopathy and was the only biomarker for myocardial fibrosis among other miRNAs (89). By genome-wide profiling of the cardiac transcriptome after MI, alteration of a large number of cardiac-specific lncRNAs was identified. These lncRNAs participate in various physiological process of cardiac cells, and therefore serve as attractive candidate biomarkers for clinical use (90). Long Intergenic NcRNA Predicting Cardiac Remodeling (LINPCR) was well identified as an efficient biomarker to predict cardiac remodeling and severity of HF (91, 92). Circulating lncRNA H19 was upregulated in decompensated right ventricle (RV) from patients with pulmonary arterial hypertension and positively correlated with RV hypertrophy and fibrosis. Importantly, a high level of miR-29 could predict long-term survival (93).

Apart from clinical outcome, ncRNAs can also be used to predict therapeutic efficacy. Upregulation of several miRNAs (miR-26-5p, miR-145-5p, miR-92a-3p, miR-30e-5p, and miR-29a-3p) in serum was associated with a better response to cardiac resynchronization therapy (94). Protective role of remote ischemic conditioning in adverse LV remodeling after MI was dependent on miR-144 (95). Sacubitril/valsartan was used to decrease pathological fibrosis and myocardial hypertrophy in HF. A study has demonstrated that treatment with sacubitril/valsartan could induce increase of specific circulating exosome containing fibrosis-related miRNAs. Signature of exosomal miRNAs after sacubitril/valsartan treatment could serve as potential biomarkers for drug response (96). Another study revealed that expression profiles of miRNAs could reflect efficiency of exercise (97) and intensive glycemic control (98) for diabetic heart disease. In addition, there were different expression patterns of miR-21 and miR-221 in CFs between failing RV and LV in a canine biventricular HF model. This may be one of the explanations that the failing RV does not respond like the LV to guideline-directed medical therapy of HF (99). Considering differences in genetic and embryonic developmental background, this result needs to be further confirmed in patients. LncRNAs also function as biomarkers for drug response. A study demonstrated that mitoquinone could ameliorate pressure overload induced cardiac fibrosis and LV dysfunction in mice, which was confirmed by improved expression level of cardiac remodeling associated lncRNAs (100).

## Therapeutic potential of non-coding RNAs for cardiac fibrosis

As mentioned above, overexpression or deletion of some ncRNAs through either their mimics or gene silencing has been proved to significantly ameliorate cardiac fibrosis and improve heart function. Notably, ncRNAs are naturally occuring regulatory molecules in cells and could target multiple genes related to cardiac fibrosis (101). Developing new technologies targeting ncRNAs seems to be a prospective choice to inhibit and reverse cardiac fibrosis.

It has developed a large number of RNA-based therapeutics, such as antisense oligonucleotides (ASO), small interfering RNA, miRNA sponges, and miRNA mimics (101). Recently, preclinical and clinical studies have demonstrated that inhibition of pro-fibrotic miRNAs at a proper time, using novel locked nucleic acid ASO, could attenuate myocardial fibrosis and give rise to better outcome in pigs or humans suffering from HF (102–104). Small molecules have great advantages to target ncRNAs because of their good solubility, bioavailability, and metabolic stability (101), such as natural compounds. Some experiments have identified potential natural compounds as anti-fibrotic drugs through downregulating harmful miRNAs *in vivo* (56, 105).

Stem cell based therapy has been at the forefront for many years, but the actual effects are limited because of low survival rates of these cells *in vivo*. Specific combination of multiple miRNAs could prevent transplanted stem cells from apoptosis *in vivo* and significantly improve their therapeutic ability (106, 107).

It is worth mentioning that regulatory mechanisms of lncRNAs in cells are complex and some lncRNAs lack interspecies sequence conservation, which makes it difficult to be targeted and carry out preclinical trials in animal models. Therefore, there are no lncRNA-targeting therapeutics for cardiac fibrosis entering clinical development so far.

Before translation into clinic, we need to avoid off-target effects and genotoxicity of ncRNA-based therapy. NcRNAs have complicated regulatory networks from various cell types to multiple organs. For example, a great deal of evidence supports the profibrotic effects of miR-21. It seems that miR-21 is one of the perfect therapeutic targets for cardiac fibrosis. But other studies have found beneficial effects of miR-21 in cardiovascular disease. MiR-21 could lower blood pressure and improve cardiac function in hypertension *via* decreasing production of reactive oxygen (8). In addition, miR-21 could prevent plaque necrosis during atherogenesis and protect CMs from apoptosis in the context of MI (108–111). Injection of AAV6-miR-199a to pigs subjected to MI could significantly promote regeneration of CMs, reduce fibrotic area, and alleviate cardiac dysfunction in early period, but sudden death occurred unexpectedly in 70% pigs under treatment because of ventricular fibrillation caused by unbalanced proliferation of CMs (112). Inappropriate inhibition of miR-21 or overexpression of miR-199a at a wrong time and dosage could be harmful to the heart.

To solve potential issues described above, cell-type-specific and organ-specific ncRNAs modulation is necessary. Exosomes, nanoparticle, and viral vector can be modified with specific ligands according to markers on the surface of different cell types and therefore provide good choice for specific delivery of AOS or mimics. Exosomes are small (30–100 nm) cell-derived membrane vesicles, which contain DNA, RNA, and protein. Exosomes can mediate communications between different cell types and therefore regulate a wide variety of local and systemic cellular processes, including cardiac fibrosis. It has been demonstrated that miRNAs in the exosomes could be delivered to cardiac tissues and improve cardiac function (96, 113–117). Many in-depth studies have revealed advantages of nanomaterials on drug delivery in recent years and an experiment has found that nanoparticle delivery of miR-29b to cardiac tissues could improve myocardial remodeling (118). Moreover, antibodies-decorated lipid nanoparticles have been used to deliver mRNAs to T cells, which could generate chimeric antigen receptor T cells *in vivo* to specifically eliminate activated CFs and improve cardiac functions (119). This attractive approach might also be used for cell-type-specific modulation of ncRNAs in cardiac tissues. Viral vector based regulation of some miRNAs could also remarkably ameliorate pathological remodeling (14, 120). A group of reported engineered AAVs could specifically deliver genes to rodent and non-human primate nervous system recently (121); developing engineered AAVs for ncRNAs delivery to CMs or CFs is one of the promising directions in the future.

## Conclusion

NcRNAs play a critical role in the development of cardiac fibrosis. Therapeutics based on ncRNAs provides a prospective direction for fibrosis-related heart diseases. Although the understanding of the relationship between ncRNAs and cardiac fibrosis has been advanced considerably in recent decades, several issues remain to be overcome. First, animal models widely used about cardiac fibrosis exhibit obvious discrepancies to corresponding human diseases (122), which might lead to contradictory results among different studies and limited translation to clinic. Second, 3p strand and 5p strand of miRNAs have distinct functions, but most studies did not distinguish 3p strand and 5p strand. Third, upstream modulators and exact cellular resources of ncRNAs remain unknown in the context of cardiac diseases. Thus, it is not enough for us to understand the functions of ncRNAs more accurately at present. Further validation of ncRNAs in the pathogenesis of cardiac fibrosis would highlight their potential as diagnostic and prognostic markers and provide a novel strategy for the treatment of cardiac fibrosis.

## Author contributions

XZ, YD, NP, LD, and ST: writing and editing. XZ and YD: revising. XZ: funding acquisition. All authors contributed to the article and approved the submitted version.
